# Complexities in Pregnancy: Diabetic Ketoacidosis Initially Thought to Be Hyperemesis Gravidarum Resulting in Early Fetal Demise

**DOI:** 10.7759/cureus.87474

**Published:** 2025-07-07

**Authors:** Jessica M Orgovan, Amanda G Sherman, Erin P Porfeli, Berk Taskin

**Affiliations:** 1 Medical School, West Virginia School of Osteopathic Medicine, Lewisburg, USA; 2 Obstetrics and Gynecology, Mon Health Medical Center, Morgantown, USA; 3 Emergency Medicine, Mon Health Medical Center, Morgantown, USA

**Keywords:** diabetes mellitus, diabetic ketoacidosis, hyperemesis gravidarum, pregnancy, spontaneous abortion

## Abstract

Hyperemesis gravidarum and diabetic ketoacidosis (DKA) can be difficult to distinguish in pregnant patients, as both conditions present with nausea, vomiting, and elevated ketones. Although uncommon, there should be a high index of suspicion for DKA in pregnant patients who exhibit these symptoms, given the potential for life-threatening maternal and fetal outcomes. Here we present a rare case of covert DKA in the first trimester of pregnancy that was initially diagnosed and treated as hyperemesis gravidarum due to the absence of a wide anion gap, which tragically resulted in fetal demise. The patient was admitted to the intensive care unit (ICU) and hospitalized for over a week but was eventually discharged in stable condition with a favorable prognosis. This report aims to contribute to clinical awareness of this metabolic complication and to emphasize the importance of prevention, early recognition, and timely management of DKA in pregnancy, along with vigilant fetal monitoring in diabetic patients.

## Introduction

Nausea and vomiting are common complications in pregnancy, with 70% to 85% of pregnant patients being affected [1]. Hyperemesis gravidarum is classified as uncontrolled vomiting, severe dehydration, electrolyte imbalance, ketonuria, muscle wasting, and weight loss that can sometimes require hospitalization [1]. This is much less common, only occurring in 0.3% to 2.3% of all pregnancies [1]. The exact cause of hyperemesis gravidarum is unknown, but symptoms usually peak at nine weeks of gestation and begin to subside around 20 weeks of gestation [1]. The treatment for hyperemesis gravidarum includes supportive care with rehydration and antiemetics; however, a thorough clinical workup is first needed to rule out other potentially life-threatening conditions. Potential diagnoses to consider include diabetic ketoacidosis (DKA), thyrotoxicosis, adrenal insufficiency, hypercalcemia, peptic ulcer disease, appendicitis, acute fatty liver of pregnancy, bowel obstruction, pyelonephritis, urinary tract infection, central nervous system disease, vestibular disease, and drug-induced vomiting [1].

DKA is an emergent complication of uncontrolled diabetes mellitus (DM) that is associated with substantial morbidity and mortality. While most cases occur in type 1 diabetes, DKA can also present in type 2 and gestational diabetes. DKA is characterized by hyperglycemia, anion-gap metabolic acidosis, and elevated ketone levels [2]. The clinical presentation of DKA manifests as symptoms of hyperglycemia, such as polydipsia, polyuria, weakness, and associated weight loss [2]. Ketonemia leads to gastrointestinal symptoms like abdominal pain, nausea, and vomiting [2]. Physical examination findings usually show signs of volume depletion, such as hypotension, tachycardia, dry mucous membranes, and decreased skin turgor [2]. Additionally, patients can present with acetone or “fruity” breath odor and rapid or deep breathing known as Kussmaul respirations [2]. Lethargy and altered mental status are often seen, and in severe cases, this may progress to loss of consciousness, respiratory compromise, and coma [2]. Physiological stressors that can trigger DKA include vomiting, acute infections, myocardial infarctions, stroke, acute pancreatitis, trauma, burns, surgery, new-onset diabetes, medication noncompliance, and inadequate dosing of insulin [2]. Medications that can precipitate DKA include glucocorticoids, beta-blockers, thiazide diuretics, sodium-glucose co-transporter 2 (SGLT2) inhibitors, and atypical antipsychotics [2]. DKA can also be induced by psychological conditions such as depression, eating disorders, and alcohol and illicit substance abuse [2].

DKA is a rare complication seen in only 0.5% to 3% of all diabetic pregnancies [3]. It is especially dangerous in pregnancy because it can develop rapidly and occur with lower or even euglycemic blood glucose levels compared to the non-pregnant population [3]. This is due to the normal physiologic changes seen in pregnancy that result in altered metabolic demands [3]. Homeostatic imbalances caused by DKA, such as severe hyperglycemia, acidosis, volume depletion, and electrolyte imbalances, pose a significant risk to a developing fetus. DKA has a fetal mortality rate between 15% and 60%, with higher incidences of fetal death correlating with maternal intensive care unit (ICU) admissions and higher serum osmolality levels [3-4]. The treatment of DKA in pregnancy is the same as in non-pregnant patients and includes careful fluid replacement therapy, insulin infusion, correction of electrolyte abnormalities, and identification and treatment of underlying precipitating causes [3-4].

## Case presentation

This is the case of a 36-year-old multiparous female patient with a past medical history significant for type 1 DM, hypothyroidism, and hypertension who presented to an outside emergency department (ED) with a chief complaint of nausea and vomiting for one day. Her symptoms progressed gradually to the point that she was not able to tolerate solids or liquids, which prompted her to seek care. She noted a positive home pregnancy test a few days prior; however, she was unsure how far along in the pregnancy she was. She admitted to taking less of her insulin because she was not able to keep anything down, including her oral prescription medications. She denied vaginal discharge, bleeding, leakage of fluid, abdominal pain or cramping, fever, chills, headaches, visual changes, chest pain, diarrhea, or dysuria. The patient shared that she had been hospitalized for DKA several times in the past and noted that her current symptoms felt similar; however, she later expressed feeling that her concerns were not being taken seriously at the time, which may have contributed to a delay in diagnosis.

The patient’s hypothyroidism and hypertension had been well managed with levothyroxine and losartan, respectively; however, she had a long-standing history of poorly managed type 1 DM despite the usage of a continuous glucose monitor as well as basal and bolus insulin injections. Her most recent hemoglobin A1C was 10.6% (nondiabetic reference range: 4%-5.6%). Her past surgical history consisted of two cesarean sections. Her obstetric/gynecologic history was significant for seven pregnancies, three term deliveries, one preterm delivery, one spontaneous abortion, one induced abortion, and three living children. She admitted to having uncontrolled blood sugars during her previous pregnancies but denied ever having been in DKA during pregnancy before. She stated that her menstrual cycles have always been irregular, and she was unsure of when the first day of her last period was. She was not currently using any form of contraception but had taken progestin birth control pills in the past. The patient’s family history was only significant for hypertension and glaucoma in her mother. She did admit to smoking half a pack of cigarettes daily for the past 21 years. She also reported occasional social alcohol consumption and weekly recreational marijuana usage but immediately discontinued both upon learning of her pregnancy.

On examination in the emergency department, the patient appeared acutely ill. She was hypertensive and tachycardic; however, she was afebrile and breathing comfortably. She initially spoke in short sentences but deteriorated to the point that she just nodded her head yes or no to questions between episodes of vomiting. Her physical examination was overall normal, including volume status, cardiopulmonary, and abdominal examinations. Comprehensive laboratory work was ordered and showed results significant for elevated beta-human chorionic gonadotropin (beta-hCG), hyperglycemia, anemia, mild ketonemia, metabolic acidosis with a normal pH, hypokalemia, gross glucosuria, and ketonuria (Table 1). DKA was initially ruled out because of the absence of a wide anion gap, and a diagnosis of hyperemesis gravidarum was made. A transvaginal ultrasound was performed, and intrauterine pregnancy was visualized; however, the gestation was too small for accurate measurements to be obtained due to early pregnancy. The patient was admitted for intravenous (IV) rehydration and IV antiemetics, in addition to attempted optimization of her subcutaneous insulin regimen and transition from losartan to labetalol for blood pressure control. Following a night of observation and resolution of vomiting, she was discharged on oral antiemetics and instructed to follow up with the maternal-fetal medicine specialist in one to two weeks to establish prenatal care as well as with her primary care physician (PCP) for the maintenance of her insulin regimen.

**Table 1:** Biochemical data from first hospitalization The patient's laboratory results from the first encounter at the emergency department confirmed pregnancy, in addition to illustrating hyperglycemia, anemia, mild ketonemia, metabolic acidosis with a normal pH, hypokalemia, gross glucosuria, and ketonuria. H: high; L: low; Beta-hCG: beta-human chorionic gonadotropin; paCO₂: partial pressure of carbon dioxide; pO₂: partial pressure of oxygen; HCO₃: bicarbonate; BUN: blood urea nitrogen; WBC: white blood cell

Lab test	Patient's values	Reference range
Glucose (mg/dL)	325 (H)	74-106
Beta-hCG (mIU/mL)	2510 (H)	<5
Venous pH	7.38	7.35-7.45
Venous paCO₂ (mmHg)	40	35-45
Venous paO₂ (mmHg)	60	60-90
Serum HCO₃⁻ (mEq/L)	19 (L)	22-30
Anion gap	10	4-12
Beta-hydroxybutyrate (mmol/L)	0.9 (H)	<0.4
Urine glucose (mg/dL)	>1,000 (H)	Negative
Urine ketones (mg/dL)	100 (H)	Negative
Lactate (mmol/L)	1.2	0.4-2.0
Potassium (mEq/L)	2.7 (L)	3.5-5.1
Sodium (mEq/L)	141	136-145
Chloride (mEq/L)	112 (H)	98-107
BUN (mg/dL)	10	9-23
Creatinine (mg/dL)	0.51 (L)	0.55-1.02
WBC count (×10³/μL)	6.7	3.7-10.5
Hemoglobin (g/dL)	10.5 (L)	11.7-15.7

**Figure 1:**
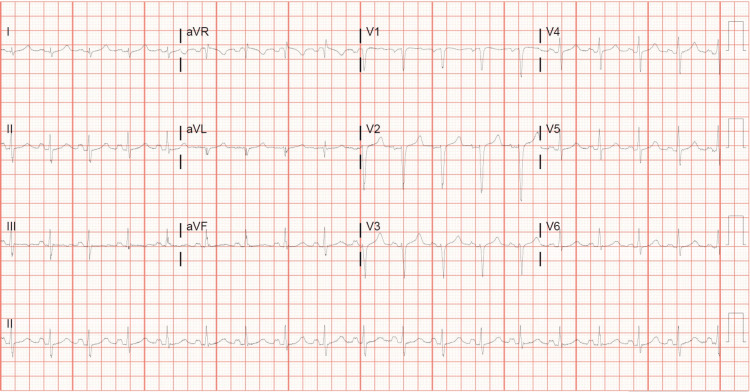
A 12-lead electrocardiogram (EKG) showing sinus tachycardia without evidence of ST-segment changes or arrhythmia.

**Figure 2:**
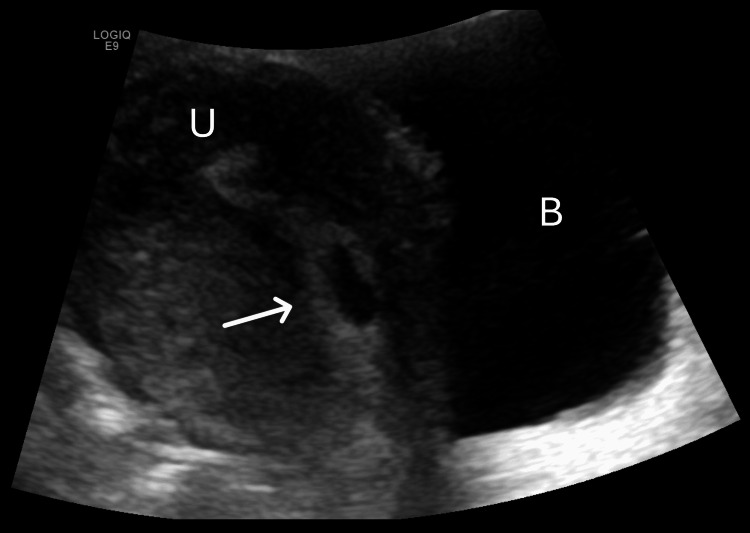
Transvaginal ultrasound, sagittal view Retained products of conception (arrow) were visualized within the lower segment of the uterus. U: uterus; B: bladder

The patient went home, and over the next day, her symptoms returned with increased severity. She was not able to tolerate anything by mouth, including the oral antiemetics she was prescribed. On the second day following discharge, she came to our institution’s ED by ambulance with unstable vital signs significant for hypertension, tachycardia, and tachypnea. She admitted to abdominal pain, shortness of breath, and what she described as "soreness" in her chest, which was exacerbated by vomiting. She denied any vaginal discharge, bleeding, leakage of fluid, headaches, or visual changes. On physical examination, she appeared lethargic and diaphoretic and was breathing rapidly. Her mucous membranes were dry, and skin turgor was decreased. Cardiopulmonary and neurological examinations were normal. On abdominal examination, she had normoactive bowel sounds, no dullness or hyperresonance to percussion, and no evidence of peritoneal irritation. There was tenderness to deep palpation in both the right and left lower quadrants as well as the suprapubic area. Comprehensive laboratory work was significant for an appropriate increase in beta-hCG levels, hyperglycemia, anemia, leukocytosis, anion gap metabolic acidosis, proteinuria, glucosuria, and ketonuria (Table 2). The patient also had a 12-lead electrocardiogram (EKG) showing sinus tachycardia without ST-segment elevations or depressions (Figure 1), and troponin levels were undetectable, effectively ruling out acute coronary syndrome. She was immediately started on IV fluid replacement therapy and IV antiemetics and given an insulin bolus. She proceeded to experience worsening abdominal pain and cramping in addition to new-onset vaginal bleeding with the passage of large clots. A transvaginal ultrasound was performed and confirmed an incomplete abortion with products of conception remaining in the lower uterine segment (Figure 2). The patient required ICU admission with an insulin drip for confirmed DKA. She was hospitalized for eight days with several complications, including acute kidney injury, acute hypoxic respiratory failure, and sepsis due to pneumonia. During this time, her beta-hCG level trended downward to less than 100 mIU/mL, and a repeat ultrasound showed complete expulsion of the uterine contents. She was eventually discharged in stable condition, with instructions to follow up with obstetrics and gynecology until her beta-hCG level was less than five. Despite the profound emotional and physical toll of her hospitalization, the patient was discharged in stable condition with supportive follow-up plans in place, and she was anticipated to make a full recovery.

**Table 2:** Biochemical data from the second hospitalization Laboratory results obtained at admission during the patient's second hospital stay showed an appropriately elevated beta-hCG level, along with hyperglycemia, anemia, leukocytosis, anion gap metabolic acidosis, proteinuria, glucosuria, and ketonuria. H: high; L: low; Beta-hCG: beta-human chorionic gonadotropin; paCO₂: partial pressure of carbon dioxide; pO₂: partial pressure of oxygen; HCO₃: bicarbonate; BUN: blood urea nitrogen; WBC: white blood cell

Lab test	Patient's values	Reference range
Glucose (mg/dL)	380 (H)	74-106
Beta-hCG (mIU/mL)	14,476 (H)	<5
Venous pH	7.01 (L)	7.35-7.45
Venous paCO₂ (mmHg)	15 (L)	35-45
Venous paO₂ (mmHg)	60	60-90
Serum HCO₃⁻ (mEq/L)	11 (L)	22-30
Anion gap	19	4-12
Urine protein (mg/dL)	30 (H)	Negative
Urine glucose (mg/dL)	500 (H)	Negative
Urine ketones (mg/dL)	80 (H)	Negative
Lactate (mmol/L)	1.44	0.4-2.0
Potassium (mEq/L)	3.7	3.5-5.1
Sodium (mEq/L)	130 (L)	136-145
Chloride (mEq/L)	100	98-107
BUN (mg/dL)	21	9-23
Creatinine (mg/dL)	1.06 (H)	0.55-1.02
WBC count (×10³/μL)	17.1 (H)	3.7-10.5
Hemoglobin (g/dL)	10.1 (L)	11.7-15.7

## Discussion

The presentations of hyperemesis gravidarum and DKA can have several features in common, including nausea, vomiting, abdominal pain, dehydration, weight loss, and a state of starvation, causing elevated ketone levels in the blood and urine [1-2]. Because of this, a high clinical suspicion and a thorough medical workup, with a holistic review of the patient, should be completed to rule out DKA as a differential when suspecting hyperemesis gravidarum. The differentiation can be even more complicated if there is no known history of diabetes, as DKA can be the first presenting symptom of a new diagnosis. This struggle has been illustrated in the reported case of an early second-trimester miscarriage, where undiagnosed type 1 DM was first presenting as DKA and initially mimicking hyperemesis gravidarum [5]. Although there is significant overlap in signs and symptoms, there should traditionally be distinctions in the laboratory workup of these two conditions. For example, a case of hyperemesis gravidarum generally presents with euglycemia, and the most common metabolic abnormality is metabolic alkalosis due to excess gastric acid loss from severe and prolonged vomiting [1]. On the other hand, DKA is expected to have hyperglycemia and the classic anion gap metabolic acidosis due to the buildup of ketone bodies [2]. However, these characteristics are not always as clear-cut due to the presence of concurrent comorbid conditions and additional acid-base disturbances.

DKA can present abnormally, especially during pregnancy, where it may present with relatively lower to euglycemic blood sugar levels [3, 6-10]. This is due to the endocrine changes seen in pregnancy, such as increased insulin resistance driven by the hormones estrogen, progesterone, cortisol, human placental lactogen (HPL), and tumor necrosis factor-alpha (TNF-α) [6]. The decline in insulin sensitivity is a physiologic mechanism that helps to provide glucose to the developing fetus and placenta, which uses copious amounts of maternal glucose as an energy source [6]. This, in turn, leads to a decrease in maternal fasting glucose levels, which results in a ketosis-prone state [6]. Pregnancy also causes an increase in minute ventilation, the amount of air that enters and leaves the lungs per minute, which results in a state of respiratory alkalosis [6]. This is usually overcome by an increase in the renal excretion of bicarbonate, ultimately leading to a reduction in the buffering capacity of the kidneys [6]. Overall, these metabolic adaptations can rapidly precipitate the onset of ketoacidosis at lower glycemic levels than those observed in diabetic patients who are not pregnant [3, 6-10].

In addition to pregnancy, the typical metabolic profile of DKA can be further skewed by additional acid-base disturbances that can complicate diagnosis and management. One of the first signs and a common symptom of DKA is vomiting; however, severe and prolonged episodes of vomiting can result in metabolic alkalosis from excessive gastric hydrogen ion loss [10-11]. This can cause elevations of the pH level, resulting in ketoalkalosis, also referred to as “masked DKA” or “alkaline ketoacidosis” [12]. Six rare cases of ketoalkalosis have been reported and attributed to nausea and vomiting in patients with type 1 DM, as well as fasting and muscular dystrophy in patients without diabetes [12]. DKA has also been documented as presenting with a normal anion gap with concurrent hyperchloremia and without any other obvious causes of hyperchloremic metabolic acidosis [13-14]. This illustrates how DKA can exist with a broad range of acid-base patterns and that the absence of an anion gap should not automatically exclude a diagnosis of DKA. In our case, the patient experienced an entire day of persistent nausea and vomiting before her initial hospital visit, during which she reduced her insulin dosage due to decreased oral intake. This combination of repeated vomiting and inadequate insulin use likely triggered the onset of DKA while simultaneously inducing metabolic alkalosis. As a result, her pH remained within the normal range, and the normal anion gap was likely due to the mild elevation of ketone bodies seen in the preliminary stages of DKA. Unfortunately, this concealed the classic diagnostic criteria for DKA in our patient, making the diagnosis less immediately apparent and prolonging treatment. In cases such as this, the consequences of delayed diagnosis can be both medically significant and deeply personal, particularly when maternal health challenges lead to fetal loss.

Our case illustrates the importance of early diagnosis and prompt treatment of DKA during pregnancy, as it is associated with substantial morbidity and mortality for both the mother and fetus. DKA is the most common cause of death in children and adolescents with type 1 DM and is responsible for around 50% of deaths in patients with diabetes less than 24 years old [2]. The insults posed on the fetus during DKA result in a mortality rate between 15% and 60%, with higher incidences of fetal death correlated to maternal intensive care unit (ICU) admission and higher serum osmolality levels [3-4]. The high rate of fetal lethality from maternal DKA has been attributed to hypovolemia, acidosis, and electrolyte imbalances experienced secondhand by the fetus. The hypovolemia and acidosis that occur during DKA lead to decreased uteroplacental blood flow and an increase in the concentration of catecholamines, ultimately causing fetal hypoxemia [3, 15-16]. Electrolyte imbalances, such as fetal hyperinsulinemia-induced hypokalemia, can cause abnormal cardiac arrhythmias seen on fetal heart rate (FHR) tracing [3, 15-16]. Studies show that rapid and intense treatment to replenish volume status, treat acidemia, and correct electrolyte abnormalities caused by DKA can normalize FHR, prolong the pregnancy, and aid in fetal survival [9, 17].

The long-term effects of ketosis on the mother and neonate have limited research, but adverse outcomes such as fetal macrosomia, congenital malformations, impaired central nervous system development, and complications during delivery have been reported [18]. These findings underscore the critical importance of preventing DKA in pregnancy in the first place to minimize potential risks to both the mother and offspring. The prevention of DKA starts with thorough pre-conceptual counseling and education on the precipitating factors, signs, and symptoms of DKA. Patients should be reminded of the importance of compliance with prenatal visits, a balanced diet, and daily monitoring of glucose levels. For those with type 1 DM, the emphasis on the importance of insulin therapy should be reiterated, and they should be made aware that the amount of insulin needed may change throughout the pregnancy. Therefore, blood sugar checks and communication with their provider are vital. For an additional parameter of prevention, the American Diabetes Association (ADA) and the American College of Obstetricians and Gynecologists (ACOG) both recommend routine monitoring for ketone bodies during pregnancy in those with type 1, type 2, and gestational diabetes [18-19]. The standards set by these organizations are to use testing strips to detect ketones in the urine when blood sugar levels reach 200 mg/dL [18-19]. Patients should be educated on the usage and interpretation of the testing strips and to contact their provider and seek care immediately if they have moderate to high amounts of ketones in their urine [18-19].

Beyond ketone monitoring, antenatal fetal surveillance testing serves as an additional indispensable measure in managing diabetes during pregnancy. The ACOG recommends initiating routine fetal testing for pregnant patients with pregestational and gestational diabetes in the third trimester with the use of shared decision-making [20]. These tests typically include repeat growth ultrasounds, nonstress tests (NSTs), and biophysical profiles (BPPs) starting at 32 weeks gestation, or earlier if complications arise [20]. Testing is repeated once or twice weekly, depending on the mothers’ glycemic control and the presence of other comorbidities [20]. These screening tools allow for early detection of potential diabetes-related abnormalities and fetal compromise, enabling timely interventions and delivery planning to mitigate adverse perinatal outcomes such as shoulder dystocia, neonatal asphyxia, and crash cesarean sections, among others [18-20].

## Conclusions

Hyperemesis gravidarum and DKA can be difficult to distinguish in pregnant patients, as they often present with overlapping symptoms. A high index of suspicion for DKA is essential, as prompt recognition and intervention are critical to preventing severe maternal and fetal complications. This case highlights the diagnostic challenges of DKA in pregnancy posed by atypical acid-base disturbances, such as concurrent metabolic alkalosis, which can obscure classic laboratory findings. Given the potential for devastating outcomes as seen in our patient, early identification and timely management are crucial. Prevention also plays a vital role and requires comprehensive patient education, diligent glucose monitoring, ketone testing, appropriate insulin adjustments, and routine antenatal fetal surveillance. By analyzing this atypical case of DKA in pregnancy, we hope to increase awareness, help clinicians recognize similar presentations, and improve maternal and fetal outcomes through both clinical vigilance and compassionate care.
